# Generative AI-enabled clinical decision support system in primary care: a pragmatic, cluster-randomized trial

**DOI:** 10.1038/s41591-026-04503-6

**Published:** 2026-06-26

**Authors:** Ambrose Agweyu, Paul Mwaniki, Vaishnavi Menon, Robert Korom, Lynda Isaaka, Conrad Wanyama, Jaspret Gill, Sarah Kiptinness, Najib Adan, Mira Emmanuel-Fabula, Richard D. Riley, Lucinda Archer, Alastair K. Denniston, Xiaoxuan Liu, Bilal A. Mateen

**Affiliations:** 1 Kenya Medical Research Institute–Wellcome Trust Research Programme, Nairobi, Kenya; 2 Keprecon, Nairobi, Kenya; 3 Department of Infectious Disease Epidemiology and International Health, London School of Hygiene and Tropical Medicine, London, UK; 4 Department of Applied Health Sciences, School of Health Sciences, College of Medicine and Health, University of Birmingham, Birmingham, UK; 5 Penda Health, Nairobi, Kenya; 6 Foundations for Appropriate Technologies in Health, Geneva, Switzerland; 7 PATH, London, UK; 8 National Institute for Health and Care Research Birmingham Biomedical Research Centre, Birmingham, UK

**Keywords:** Health care, Translational research, Developing world

## Abstract

Rigorous evidence on the performance of large language models (LLMs) in real-world, low-resource clinical settings remains limited. Here we conducted a pragmatic, cluster-randomized trial in 16 primary care facilities in Kenya. Clinical officers were randomized to use the electronic medical record with or without LLM assistance. The primary outcome was an expert-adjudicated composite of treatment failure events experienced within 14 days of enrollment. Between 22 April and 16 July 2025, 9,691 patients were enrolled, overseen by 103 clinical officers (52 in the LLM-assisted arm and 51 in the control arm). Treatment failure occurred in 102/4,693 patients (2.2%) in the intervention arm and 94/4,654 (2.0%) in the control arm (adjusted odds ratio 0.77, 95% confidence interval 0.55 to 1.08, *P* = 0.13). The primary outcome did not differ significantly between groups. No serious adverse events were judged related to the intervention, and independent review of the adverse events did not identify a safety signal. In this trial, LLM assistance was safe but did not reduce treatment failure within 14 days and any benefit, if present, is probably modest.

Pan-African Clinical Trials Registry: 202502499779176.

## Abstract (web summary)

A pragmatic cluster-randomized trial found that ChatGPT-4o-assisted decision support in Kenyan primary care facilities did not significantly reduce 14-day treatment failure over usual care.

## Main

Primary care facilities manage a wide range of acute and chronic conditions and serve as the foundation for continuity of care, coordination across health system levels and equitable service delivery^1,2^. In sub-Saharan Africa, these facilities operate under acute workforce constraints: the region has approximately 0.3 physicians per 1,000 population, less than 10% of the Organisation for Economic Co-operation and Development average (3.9 per 1,000)^3,4^. Owing to this physician shortage, in Kenya and many other countries, primary care is often delivered by clinical officers—mid-level practitioners who complete a 3-year diploma in clinical medicine^5^. These primary care providers often face complex diagnostic and treatment decisions without access to senior consultation^6,7^. This can lead to inconsistent adherence to guidelines, diagnostic errors, inappropriate treatment and, ultimately, unfavorable patient outcomes^8,9^. Crucially, 60% of deaths from conditions amenable to healthcare in low- and middle-income countries (LMICs) occur among individuals who had already accessed the health system, indicating that the quality, not just the availability of care, is a critical challenge in LMICs^7,9^.

Large language models (LLMs) have demonstrated the ability to interpret clinical information and produce context-appropriate recommendations consistent with clinical guidelines. Vignette-based comparisons suggest that LLMs can match or exceed provider performance on some diagnostic and triage tasks^10–12^. This potential of LLMs to improve the quality of care delivered by frontline healthcare workers has also been demonstrated using in silico methods in several low-resource settings^13–15^. However, prospective interventional evidence from real-world clinical studies, particularly in LMICs, remains limited^16–19^.

Experience with traditional rule-based digital tools embedded in electronic medical record (EMR) systems, in LMICs, suggests that, although such systems can enhance adherence to clinical protocols, their impact has been limited by rigid logic, heavy data-entry requirements and weak integration with routine clinical workflows^20^. Generative LLMs differ in that they can reason across unstructured data, emulate dialog and adapt recommendations to nuanced presentations. However, because most training data are largely derived from high-income health systems, these models may be poorly calibrated to the epidemiology, documentation styles and resource constraints of LMICs^21^. Their generative nature also raises concerns about accuracy, hallucinated content and bias^22,23^. Empirical evidence is therefore needed to determine whether integrating LLMs into routine primary care can enhance clinical reasoning, documentation and clinical outcomes while safeguarding patient safety.

In this study, we conducted a cluster-randomized trial to explore whether a generative artificial intelligence (AI)-powered (that is, LLM-based) clinical decision support system embedded within a cloud-based EMR, that is, ‘the AI consult’^24,25^, can improve the quality of care delivered by clinical officers across a network of 16 primary care facilities operated by Penda Health in Nairobi and Kiambu counties in Kenya. The primary aim was to assess the effect of the intervention on patient treatment failure, with additional evaluation of clinical documentation, prescribing practices, patient satisfaction and safety outcomes. Given that the intervention was delivered at the level of the clinical officer within routine workflows, individual-level randomization was not feasible, as clinicians could not realistically alternate between intervention and control conditions without risk of within-clinician contamination. We therefore used design with randomization at the level of the clinical officer, with patients nested within clinicians and health facilities.

## Results

### Patient disposition

**Fig. 1:**
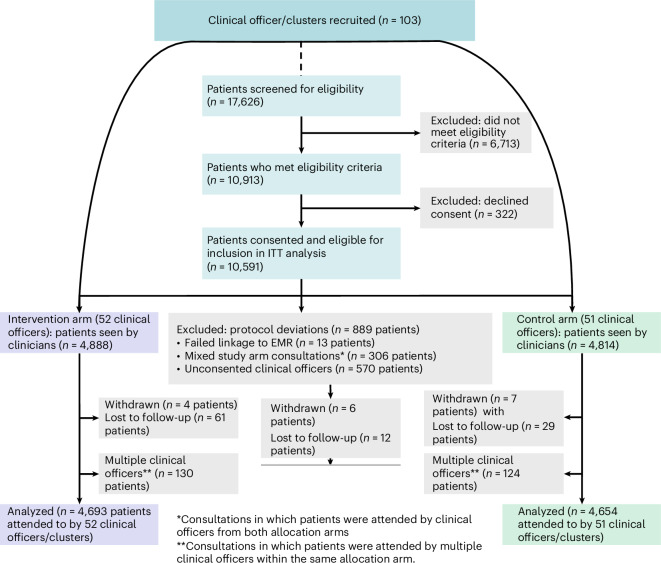
CONSORT flow diagram of participant and cluster progression throughout the trial. The diagram shows the number of patients screened, enrolled and allocated according to the clinical officer (cluster) assignment, and those included in the ITT analysis. Reasons for exclusion after enrollment included withdrawal of consent, loss to follow-up and protocol deviations (including encounters managed by clinicians assigned to a different study arm). Cluster randomization occurred at the level of the clinical officer, and all participants were analyzed according to the allocation of the treating clinician.

Between 22 April and 16 July 2025, 17,626 patients were screened for eligibility. Clinical officer clusters (*n* = 103; 52 in the LLM-assisted arm and 51 in the control arm) were randomized to either the intervention or control arm, and 9,702 eligible patient encounters were included and analyzed according to the allocation of the treating clinical officer. After removing individuals on the basis of withdrawals (*n* = 11; 4 in the intervention arm and 7 in the control arm), loss to follow-up (*n* = 90; 61 intervention and 29 control) and encounters affected by protocol nonadherence leading to potential exposure or allocation misclassification (*n* = 254; 130 intervention and 124 control), the primary analysis included 4,654 control and 4,693 intervention encounters (Fig. 1). The trial was completed as planned after reaching the prespecified sample size and follow-up period. Baseline demographic and clinical characteristics were similar across study groups (Table 1). Most patients (5,658/9,347; 61%) were aged 18–55 years, and 5,271 (56%) were female. The most common presenting diagnoses were febrile or infectious illnesses (5,585; 60%). The distribution of clinical officer characteristics was similar between groups (Supplementary Table 1). The mean per-patient LLM cost in the intervention arm, based on the average number of tokens used per consultation, was US$0.04 (95% confidence interval (CI) 0.04 to 0.04).

**Table 1:** Patient characteristics by study arm

Characteristic	Overall *N* = 9,347	Control *N* = 4,654	Intervention *N* = 4,693
Age			
<1 year	491 (5.3%)	231 (5.0%)	260 (5.5%)
1–4 years	1,665 (18%)	828 (18%)	837 (18%)
5–17 years	1,397 (15%)	703 (15%)	694 (15%)
18–55 years	5,658 (61%)	2,820 (61%)	2,838 (60%)
>55 years	136 (1.5%)	72 (1.5%)	64 (1.4%)
Sex			
Female	5,271 (56%)	2,622 (56%)	2,649 (56%)
Male	4,076 (44%)	2,032 (44%)	2,044 (44%)
Diagnosis*			
Cardiovascular	495 (5.3%)	244 (5.2%)	251 (5.3%)
Dermatologic	1,273 (14%)	615 (13%)	658 (14%)
Ear, nose and throat; dental; and ophthalmologic	4,223 (45%)	2,100 (45%)	2,123 (45%)
Febrile/infectious	5,585 (60%)	2,833 (61%)	2,752 (59%)
Gastrointestinal	2,624 (28%)	1,270 (27%)	1,354 (29%)
Genitourinary and reproductive	1,855 (20%)	931 (20%)	924 (20%)
Musculoskeletal	1,392 (15%)	724 (16%)	668 (14%)
Neurologic and psychiatric	1,242 (13%)	619 (13%)	623 (13%)
Respiratory	3,300 (35%)	1,710 (37%)	1,590 (34%)
Unspecified/other	34 (0.4%)	15 (0.3%)	19 (0.4%)
Health facility			
CBD Kimathi Street	326 (3.5%)	168 (3.6%)	158 (3.4%)
Embakasi	483 (5.2%)	238 (5.1%)	245 (5.2%)
Githurai 45	417 (4.5%)	158 (3.4%)	259 (5.5%)
Kahawa West	613 (6.6%)	391 (8.4%)	222 (4.7%)
Kangemi	678 (7.3%)	441 (9.5%)	237 (5.1%)
Kasarani	370 (4.0%)	208 (4.5%)	162 (3.5%)
Kawangware	353 (3.8%)	24 (0.5%)	329 (7.0%)
Langata	285 (3.0%)	221 (4.7%)	64 (1.4%)
Lucky Summer	565 (6.0%)	406 (8.7%)	159 (3.4%)
Mathare North	395 (4.2%)	92 (2.0%)	303 (6.5%)
Pipeline	675 (7.2%)	585 (13%)	90 (1.9%)
Sunton	369 (3.9%)	124 (2.7%)	245 (5.2%)
Tassia Kwa Ndege	650 (7.0%)	236 (5.1%)	414 (8.8%)
Umoja-phase II	646 (6.9%)	238 (5.1%)	408 (8.7%)
Umoja 1	1,033 (11%)	723 (16%)	310 (6.6%)
Zimmerman	1,489 (16%)	401 (8.6%)	1,088 (23%)

*Percentages sum to more than 100% because individual patients could have multiple diagnoses.

### Primary outcome

**Extended Data Fig. 1:**
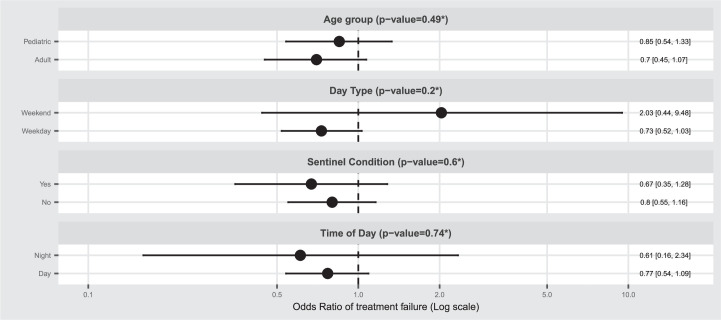
Subgroup effects on treatment failure. Subgroup analyses of the primary outcome (14-day treatment failure) were conducted across predefined categories, including age group (pediatric, n = 3,553; adult, n = 5,794), day type (weekend, n = 561; weekday, n = 8,786), sentinel condition status (yes, n = 1,098; no, n = 8,249), and time of presentation (night, n = 356; day, n = 8,991). Points and error bars represented aORs with 95% confidence intervals, derived from mixed-effects logistic regression models including interaction terms between treatment and subgroup variables. P values are based on two-sided Wald tests for the interaction terms and are not adjusted for multiple testing. Models included random effects for clinical officer and health facility to account for clustering. Estimates should be interpreted cautiously given limited precision in some subgroups due to low event rates.

**Extended Data Fig. 2:**
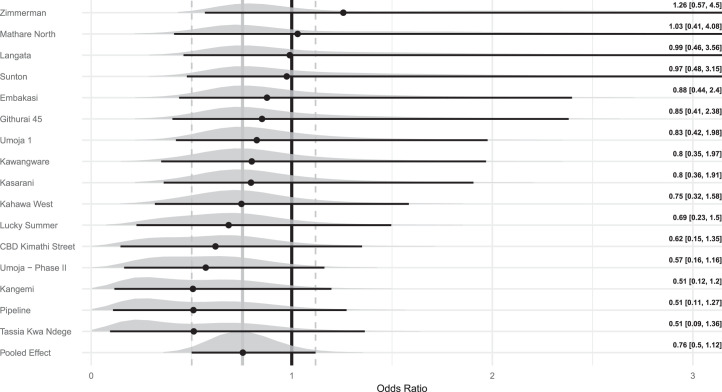
Site-level and pooled estimates for treatment failure. Forest plot showing site-specific and pooled estimates of the effect of the intervention on treatment failure. Estimates were obtained using a one-stage individual participant data (IPD) Bayesian multilevel model with a Bernoulli–logit likelihood. The model included a fixed treatment effect, hospital-level random intercepts, hospital-level random treatment slopes (to allow for heterogeneity in treatment effects), and clinician-level random intercepts to account for clustering by provider. Weakly informative priors were specified for all parameters. Points represent posterior ORs and horizontal error bars represent 95% credible intervals. Site-level estimates are presented alongside the pooled effect. Between-site heterogeneity is summarized by the posterior distribution of the standard deviation of the random treatment effects. τ = 0.22; 95% CrI, 0.01–0.67. Hospital sample sizes (n) were: CBD Kimathi Street 326; Embakasi 483; Githurai 45 417; Kahawa West 613; Kangemi 678; Kasarani 370; Kawangware 353; Langata 285; Lucky Summer 565; Mathare North 395; Pipeline 675; Sunton 369; Tassia Kwa Ndege 650; Umoja - Phase II 646; Umoja 1 1,033; Zimmerman 1,489.

Treatment failure occurred in 94 patients (2.0%) in the control group and 102 (2.2%) in the intervention group (adjusted odds ratio (aOR) 0.77, 95% CI 0.55 to 1.08, *P* = 0.13, in both the intention-to-treat (ITT) and per-protocol analysis; Table 2). Additional adjustments for encounter-specific variables (that is, sex, age, time of day and day of week) yielded similar results (aOR 0.72, 95% CI 0.50 to 1.03, *P* = 0.07). Relatedly, there was no evidence of variation in the effect of the intervention within those subgroups (Extended Data Fig. 1). Across the 16 facilities, the pooled odds ratio (OR) was 0.76 (95% credible interval (CrI) 0.50 to 1.12). Site-specific point estimates were largely consistent in direction, favoring the intervention, for 14 facilities, although individual-site intervals were wide. Between-site heterogeneity was low (*τ* = 0.22, 95% CrI 0.01 to 0.67) (Extended Data Fig. 2). The population-average risk of treatment failure corresponded to five fewer treatment failures per 1,000 patients treated (mean risk difference of −0.005, 95% CrI −0.013 to 0.001).

**Table 2:** Treatment failure by study arm (intention-to-treat and per-protocol analyses)

Analysis	Characteristic	Failure, *n* (%)	No failure, *n* (%)	aOR	95% CI	*P*value
ITT (adjusted for facility and clinical officer)	Control	94 (2.0)	4,560 (98.0)			
	Intervention	102 (2.2)	4,591 (97.8)	0.77	0.55 to 1.08	0.13
Per protocol (adjusted for facility and clinical officer)	Control	93 (2.0)	4,528 (98.0)			
	Intervention	102 (2.2)	4,591 (97.8)	0.77	0.55 to 1.08	0.13
ITT (cluster and covariate adjusted*)	Control	94 (2.0)	4,560 (98.0)			
	Intervention	102 (2.2)	4,591 (97.8)	0.72	0.50 to 1.03	0.07

*Adjusted for clustering by clinical officer and facility (random intercepts) and for patient age, sex, diagnosis, time of consultation (day versus night; weekday versus weekend) and clinical officer experience (years since qualification and tenure at Penda Health). ITT, intention to treat.

### Secondary outcomes

#### Clinical documentation quality

**Fig. 2:**
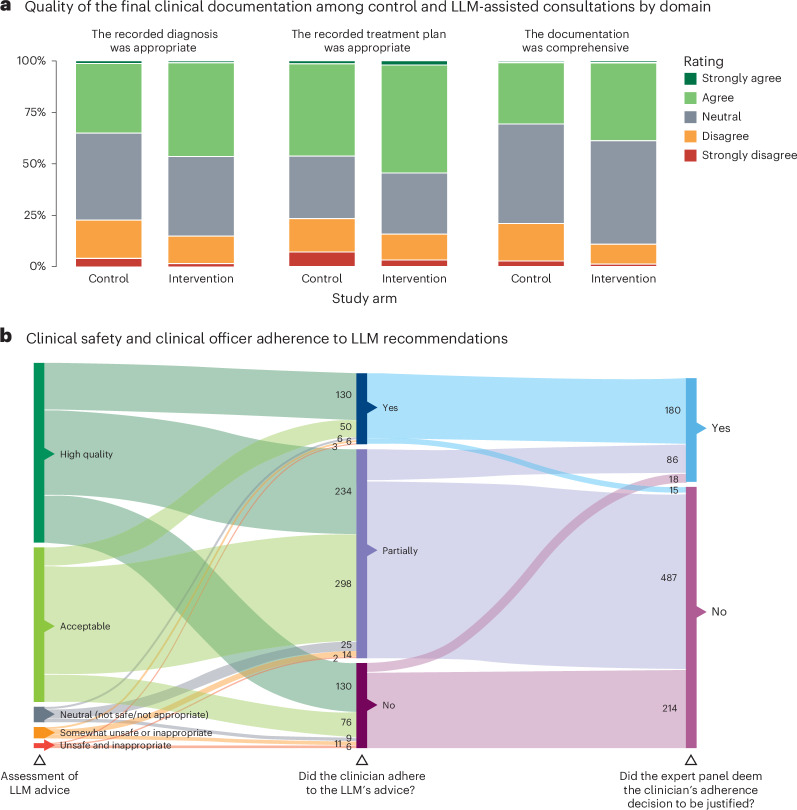
Effect of the intervention on documentation quality and clinical safety. **a**, The distribution of expert ratings of documentation quality for encounters managed with (intervention) and without (control) LLM-assisted decision support. Stacked bar charts show the proportion of encounters across ordered categories for appropriateness of diagnosis, adequacy of treatment planning and comprehensiveness of clinical documentation. **b**, A Sankey diagram illustrating clinical decision-making pathways for encounters generating high-severity (red) alerts. Flows represent sequential transitions from the quality of LLM advice, to clinician adherence to that advice (full, partial or none) and finally to expert panel assessment of whether the clinician’s decision to adhere or not adhere was clinically justified. The width of each flow is proportional to the number of encounters, with counts provided for each pathway.

Among the 2,000 encounters reviewed for documentation quality, clinical officers using LLM assistance produced higher-quality notes across all domains (Fig. 2a). Compared to the control arm, they were more likely to record an appropriate diagnosis (aOR 1.74, 95% CI 1.28 to 2.36, *P* < 0.001), a comprehensive clinical note (aOR 1.68, 95% CI 1.24 to 2.27, *P* < 0.001) and an appropriate treatment plan (aOR 1.71, 95% CI 1.25 to 2.34, *P* < 0.001).

#### Clinical safety

Out of 1,000 LLM outputs associated with red alerts (prompted by the LLM’s interpretation of the documented information being incorrect/harmful), which were reviewed by the expert panel, 494 (49.4%) were rated as definitely safe and appropriate, and 424 (42.4%) as mostly safe and appropriate. In addition, 40 (4.0%) were considered neutral (neither unsafe nor inappropriate), while 31 (3.1%) were judged somewhat unsafe or inappropriate, and 11 (1.1%) as unsafe and inappropriate. Clinicians fully adhered to the LLM’s advice in 195 encounters (19.5%), partially adhered in 573 encounters (57.3%) and did not adhere in 232 encounters (23.2%). The expert panel judged clinician decisions to be clinically justified in 284 encounters (28.4%) and not justified in 716 encounters (71.6%) (Fig. 2b).

#### Sentinel conditions

**Fig. 3:**
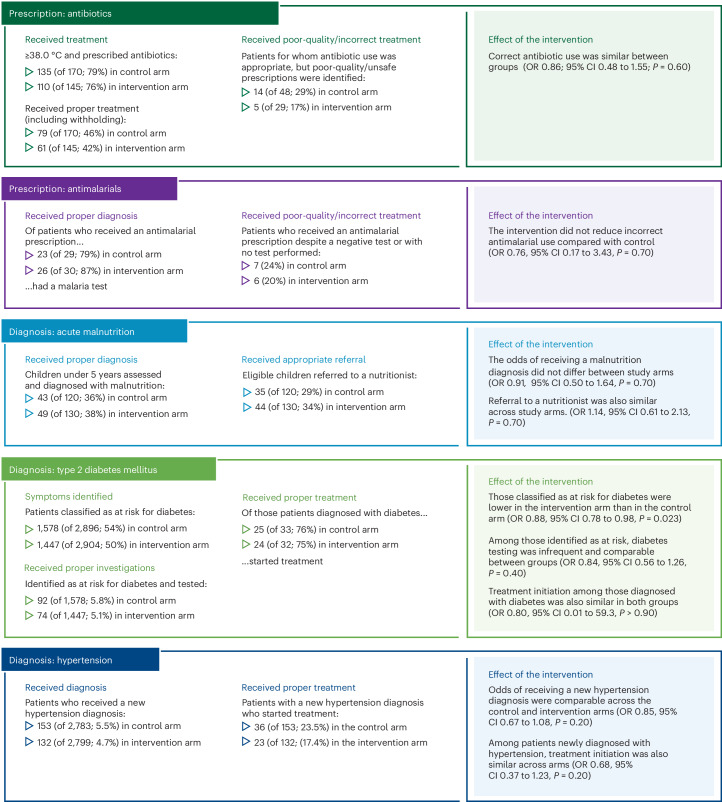
Outcomes for prescribing practices and sentinel conditions. Summary of secondary outcomes comparing intervention and control arms across prescribing practices (antibiotic and antimalarial use) and the identification and management of sentinel conditions (hypertension, type 2 diabetes mellitus and severe acute malnutrition). For each outcome, aORs with 95% CIs are presented alongside corresponding counts and proportions in each study arm. Estimates were derived from mixed-effects logistic regression models accounting for clustering by clinical officer and health facility, and *P* values were calculated using two-sided Wald tests with no adjustment for multiple testing.

There was no evidence of differences between study arms in prescribing outcomes, including correct antibiotic use (aOR 0.86, 95% CI 0.48 to 1.55) and incorrect antimalarial prescribing (aOR 0.76, 95% CI 0.17 to 3.43). Similarly, there were no differences in the diagnosis or management of hypertension in adults, including new diagnosis (aOR 0.85, 95% CI 0.67 to 1.08) and treatment initiation (aOR 0.68, 95% CI 0.37 to 1.23), or acute malnutrition in children, including diagnosis (aOR 0.91, 95% CI 0.50 to 1.64) and referral to a nutritionist (aOR 1.14, 95% CI 0.61 to 2.13). However, individuals were more likely to be identified as being at risk of type 2 diabetes mellitus in the control arm than in the intervention arm (aOR 0.88, 95% CI 0.78 to 0.98, *P* = 0.023), although treatment initiation rates among those eventually diagnosed were similar (Fig. 3).

#### Patient satisfaction

**Extended Data Fig. 3:**
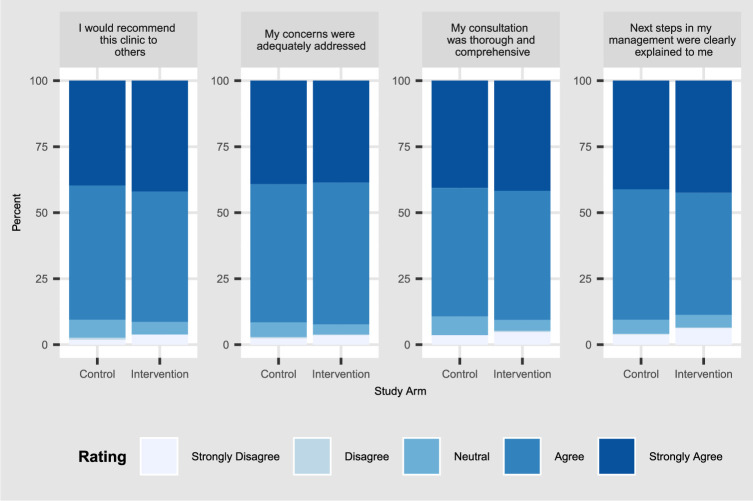
Patient-reported satisfaction by study arm. Distribution of patient-reported satisfaction scores across study arms based on exit interviews. Responses are summarized as median scores for 5-point Likert scales by study arm and presented as stacked bar charts showing proportions summing to 100%. Satisfaction domains include likelihood of recommending the clinic, adequacy of addressing concerns, thoroughness of the consultation, and clarity of explanation of next steps.

Among 826 patients who completed the satisfaction survey, 411 were in the control group and 415 in the intervention group. Demographic characteristics were similar across study arms (Supplementary Table 2). Satisfaction scores were identical at the group level (median 4.0, interquartile range (IQR) 4.0–5.0). The likelihood of reporting high satisfaction was similar in both groups (aOR 1.02, 95% CI 0.70 to 1.49, *P* > 0.9). Perceptions of consultation duration were comparable across arms: 784 respondents (95%) reported that the consultation length was ‘just right’, 20 (2.4%) indicated it was too short and 22 (2.7%) that it was too long (Extended Data Fig. 3). Post hoc analysis of median consultation time was 11 min in both arms (IQR 7–17 min in the control arm, 8–17 min in the intervention arm, 95% CI −1.0 to 0.00, *P* = 0.031).

### Safety

Across the 16 study sites, 33 serious adverse events (27 hospitalizations and 6 deaths) occurred during the study. Independent review confirmed appropriate management and no causal link to the intervention. Post hoc exploratory analysis of the composite outcome of death or hospitalization (that is, all serious adverse events), which occurred in 17 participants (0.4%) in the control arm and 14 participants (0.3%) in the intervention arm, did not significantly differ (aOR 0.77, 95% CI 0.30 to 1.94, *P* = 0.60).

### Protocol deviations and violations

A total of 921 protocol deviations and violations were documented across the 16 study sites. Protocol deviations (*n* = 876; 8.3% of recruited encounters) were mainly operational and included encounters involving unconsented clinical officers (*n* = 570; 5.4%) or more than one clinical officer (*n* = 306; 2.9%), reflecting routine workflow constraints, patient preferences, shift changes and follow-up reviews. An additional 13 encounters (0.1%) could not be linked to an EMR record. Of these, 254 encounters were excluded from the primary analysis because protocol nonadherence created potential exposure or allocation misclassification. Protocol violations (*n* = 45; 0.4%) resulted from a temporary EMR configuration error that inadvertently enabled AI Consult access among some control-arm clinical officers; the issue was promptly identified and corrected. A detailed listing of adverse events, protocol deviations and violations is provided in the study repository^26^.

### Post hoc analyses

Post hoc analysis of per-patient spending was similar between the study arms for almost all medication categories (Supplementary Table 3). Notably, antibiotics accounted for the largest average share of spending at US$3.85 in the control arm and US$3.71 in the intervention arm (exchange rate: 1 US dollar = 130 Kenya Shillings). In adjusted multilevel linear regression accounting for clinical officer and facility effects, antibiotic-related costs were lower in the intervention arm (mean difference US$−0.15, 95% CI −0.25 to −0.04) (Supplementary Table 4).

## Discussion

In this large-scale pragmatic randomized controlled trials of a generative LLM embedded in routine clinical workflows across the full spectrum of primary care, we found similar rates of 14-day treatment failure between groups, extending emerging evidence from recent randomized evaluations in other clinical settings^18,27^. The estimated effect corresponded to between 13 fewer and 1 additional treatment failures per 1,000 patients, indicating that any true effect, if present, is likely to be modest. Interpretation of the primary outcome should consider both the magnitude and precision of the estimated effect. The observed event rate was lower than anticipated, resulting in limited precision for detecting modest effects. Nonetheless, the findings provide bounded inference: large clinically meaningful effects are unlikely, while smaller effects cannot be ruled out. This pattern probably reflects the complexity of clinical outcomes in primary care, which are influenced by a range of broader contextual factors than just the care provided in clinic^9,28–30^, as well as the fact that the trial was powered for a larger effect size than the one observed. That said, there is strong evidence that the intervention improved the quality of clinical documentation and care (as demonstrated by improved diagnostic reasoning and appropriate treatment planning), reduced antibiotic-related costs and was generally safe. However, the trial was not powered to detect rare severe adverse events, and the absence of observed differences between groups should not be interpreted as evidence of safety equivalence. While no intervention-related safety concerns were identified, the data provide limited precision regarding rare harms, and uncertainty remains without a prespecified noninferiority or safety framework.

The intervention was implemented as a workflow-integrated decision support tool that generated recommendations automatically during routine documentation, without requiring clinicians to actively initiate its use. Clinicians retained autonomy to accept, modify or disregard the system’s suggestions, and no additional incentives or enforcement mechanisms were introduced to promote uptake. As a result, the trial evaluates effectiveness under routine care conditions rather than efficacy under enforced use. While variable engagement with the system may have attenuated observable effects on clinical outcomes, this design reflects real-world implementation conditions and supports the external validity of the findings.

While objective clinical endpoints such as hospitalization and death (that is, serious adverse events) are ultimately the most meaningful measures of impact, these events are rare in primary care. Post hoc power simulations based on the initial results reported indicated that detecting modest differences in rare clinical events would require substantially larger sample sizes (for example, >100,000 patients) than those feasible in the present study (Sam Waton & Jishnu Das, personal communication, 25 July 2025). Given the logistical and resource implications, and following discussion among the relevant stakeholders, a decision was made not to extend the trial. As this study exemplifies, there remains an open question around what is the most appropriate primary outcome for evaluating a general-purpose technology (for example, an LLM-based clinical decision support (CDSS)) in a broad field (for example, primary healthcare), where metrics specific to any individual disease do not sufficiently capture the value proposition, and objective patient-level impacts require a scale of intervention that is often infeasible.

The improvement in process outcomes observed aligns with findings from both controlled and real-world studies^31^. In a 40,000-encounter quality-improvement study conducted within the same primary care network as the present trial, an almost identical tool produced measurable gains in documentation completeness and adherence to clinical standards, which, in turn, mediated reductions in diagnostic and treatment errors^32^. Similarly, findings from a pilot trial in Japan reported that provider-in-the-loop note generation using an LLM improved documentation quality across all domains compared to provider-only records, while maintaining accuracy and efficiency^33^. Assuming previously described theories of change hold^34^, such incremental process improvements may ultimately translate into meaningful gains for patient outcomes^35^.

The intervention did not change antibiotic prescribing rates among febrile patients. This may be explained by how deeply ingrained antibiotic prescribing for fever is in clinical practice^36,37^, or the small size of this subgroup. That said, the observed (statistically significant) savings could be due to the increased power afforded by analyzing all trial participant data, and/or to the use of cheaper antibiotics (while maintaining a similar rate of appropriate prescriptions). This is noteworthy, as at the health-system level, many governments are working to advance universal health coverage amid tightening fiscal space and declining donor support. In the trial population alone, the direct per-patient savings from reduced antibiotic prescribing exceeded the per-patient cost of running the LLM, suggesting that such tools can generate net savings at the operational level, even before broader system or clinical benefits are considered; there is probably more nuance to consider when evaluating whether the technology is truly cost-saving once the total cost of ownership is calculated, but this is a positive signal nonetheless. The lower proportion of patients classified as at risk of type 2 diabetes in the intervention arm may reflect improved detection of individuals whom the LLM had already identified as having established diabetes. Thus, the intervention appears to have shifted a subset from the ‘at-risk’ category into known (pre-existing) diagnosis, a reclassification that may yield downstream efficiencies and cost savings by reducing unnecessary screening and assessments.

Finally, patients did not report a difference in satisfaction between arms, despite a slightly longer consultation duration in the LLM arm. Evidence from a parallel mixed-methods evaluation within the same network provides insight into why this might be—clinical officers viewed the ‘AI Consult’ as a complementary aid that improved diagnostic reasoning and thoroughness while preserving autonomy and rapport with patients^25^. In combination with the user interface never being exposed to the patient, it is understandable why they did not perceive a difference in the consultation. Such findings reinforce that when well-integrated into clinical workflows, LLM-based decision-support systems can strengthen the quality of care without diminishing the human elements essential to patient trust and satisfaction^38^. However, sustained reliance on automated reasoning could also erode providers’ skills^39–41^. More research is required to understand which deliberate design choices promote critical reasoning and potentially even upskilling, while avoiding cognitive offloading in a manner conducive to deskilling.

The study had the following limitations: although the trial was randomized at the level of the clinical officer, it was conducted within shared facility environments, and informal exchange of clinical reasoning between clinicians could not be fully prevented. However, the LLM-assisted interface was accessible only to clinical officers assigned to the intervention arm through secure, role-based login credentials within the EMR, which helped limit cross-arm exposure. Nevertheless, any residual contamination would be expected to bias results toward the null, potentially attenuating observable differences between groups.

Conducting the trial within a single private network of urban clinics in Nairobi may limit generalizability to rural, periurban or public-sector settings with different patient populations, staffing patterns and infrastructure. Because clinicians were randomized within shared facilities, informal exchange of clinical approaches between providers could not be fully excluded, which may have reduced contrast between groups and biased estimates toward the null. It is also possible that the effect of the LLM was attenuated by the relatively high baseline standards of care within the study network. Penda Health operates a structured quality-improvement framework with regular clinical audits, peer review and performance feedback through its EMR. These features probably narrowed the margin for measurable improvement compared to less digitized or lower-resourced environments, where the same intervention might yield greater benefit. As with all AI interventions, performance is tied to the specific model version and data distribution. Given the rapid pace of model evolution, newer versions are likely to show improved reasoning, safety filtering and bias control; our results should therefore be viewed as a temporal benchmark rather than a fixed estimate of capability. Finally, the 14-day follow-up period may have been too short to capture downstream effects such as reduced errors, improved continuity of care or operational efficiencies.

In summary, the intervention did not reduce short-term treatment failure, and no safety concerns were identified. Larger or adequately powered studies are needed to determine whether modest clinical benefits exist with greater precision.

## Methods

### Design

We conducted a pragmatic, multicenter, parallel-group cluster-randomized controlled trial across a network of 16 primary care facilities operated by Penda Health in Nairobi and Kiambu counties in Kenya, comparing routine clinical officer-led consultations supported by an LLM to those conducted without LLM assistance. Randomization occurred at the level of the clinical officer, who constituted the unit of clustering, with patients nested within clinical officers and facilities.

### Participants

Eligible clinical officers were those registered with the Clinical Officers Council of Kenya, actively providing outpatient care within participating facilities, and willing to use the EMR system for all consultations during the study period. Clinical officers who were not providing clinical care during the study period or who declined participation were excluded. Written informed consent was obtained from clinical officers before enrollment.

All patients whose consultation was led by a participating clinical officer during the study period were considered for inclusion. Eligibility was assessed by research assistants, who also invited patients to participate and obtained written informed consent before the consultation. Patients under 18 years of age attending for any reason were eligible, with those aged 12–18 years providing assent in addition to guardian consent. Adults or children attending for nonacute or planned wellness visits (such as weight checks, vaccinations or routine antenatal care), those unable to provide informed consent owing to impaired mental capacity, those unwilling or unable to be contacted for follow-up, and those requiring immediate emergency stabilization or referral at the time of screening were excluded.

### Interventions

In both study arms, clinical officers used the same EMR system for documentation and order entry. In the intervention arm, clinical officers had access to a custom-built LLM-based CDSS feature (called ‘AI Consult’, version 2.0), which was embedded within the EMR. The system used GPT-4o (May 2025 release) with temperature 0.1, top-p 1.0 and 1024-token response/maximum output limit. During each patient encounter, the underlying LLM (GPT-4o) analyzed information (including all structured and free-text data fields, and excluding patient identifiers) entered by the clinical officer and generated tailored diagnostic and therapeutic guidance. Clinical officers were not required to initiate a separate query. The system was initialized through structured system prompts that defined its clinical role, scope and constraints, to support alignment with Kenyan national treatment guidelines and the broader healthcare context, while retaining generative flexibility within these boundaries. The color-coded alert logic was implemented through a version-controlled prompt, specifying explicit severity thresholds, rule-based criteria and constrained output formatting (i.e. a JavaScript Object Notation schema)^26^. Although outputs were generated via LLM inference, the use of fixed instructions, explicit severity definitions and few-shot examples constrained model behavior and supported reproducibility.

In the intervention arm, feedback was displayed using a three-tier visual signal, with green indicating no issues, yellow indicating minor issues and red indicating critical concerns, to guide provider attention. The EMR interface allowed clinicians to review the LLM-generated suggestions and selectively incorporate elements into the clinical documentation, including through copy-assisted entry where appropriate. Clinical officers retained autonomy over clinical assessment, documentation, diagnosis, prescribing and referral decisions, and could accept, modify or disregard the system’s suggestions.

Clinical officers randomized to the control arm provided routine care using the same EMR system for documentation, clinical review and order entry, but with the AI Consult 2.0 feature disabled. Clinical officers were expected to follow routine clinical guidelines and practice standards irrespective of study allocation, and no additional incentives related to guideline adherence or documentation were introduced as part of the trial. The control arm therefore reflected usual care conditions within the participating facilities, including access to routine information resources available in clinical practice.

### Randomization, allocation and blinding

Randomization occurred at the provider (clinical officer) level to mitigate potential learning effects that might influence patient outcomes. Block randomization was implemented by the study statistician, with variable block sizes of four, six or eight. Clinical officers remained assigned to their randomized study arm throughout the study period and across routine clinical shifts.

Patients were assigned to study arms according to the allocation of the clinical officer managing their consultation, consistent with the cluster-randomized design, and all patients within intervention clusters were considered fully exposed to the intervention. Within the EMR, AI Consult 2.0 was activated for clinicians randomized to the intervention arm and disabled for clinicians randomized to the control arm. Because clinicians worked independently within consultation rooms, the risk of contamination between clinicians within the same facilities was considered low.

Blinding of clinical officers was not possible because those assigned to the intervention arm interacted directly with the LLM functionality within the EMR. Participating patients remained blinded to allocation throughout the study. Research assistants conducting patient satisfaction interviews and day 3 and day 14 follow-up assessments were also blinded to participant allocation. Clinical officers retained full autonomy over clinical decision-making irrespective of study allocation and could disregard or follow AI recommendations at their discretion.

### Outcomes

The primary outcome was treatment failure within 14 days of the index consultation. Treatment failure was defined as re-presentation to primary care with unresolved symptoms, unplanned escalation to higher-level or emergency care, or a safety or adverse event, including delayed or missed referral, inappropriate prescription, missed diagnosis, life-threatening event or death. Components involving clinical judgment were defined using prespecified criteria and standardized during training of the expert panel before adjudication. Outcome data were collected by research assistants (blinded to clinical officer allocation) in follow-up telephone calls on days 3 and 14 after the initial visit. For participants who were unreachable on initial attempts, additional calls were made daily until contact was achieved or until 2 weeks after the end of follow-up for the last enrolled participant.

Secondary outcomes included the quality of clinical documentation (assessed for appropriateness of diagnosis, comprehensiveness and adequacy of the treatment plan), the management of sentinel conditions (hypertension, type 2 diabetes and malnutrition), the appropriateness of antibiotic and antimalarial prescriptions, and patient satisfaction. Following completion of the consultation, a subset of 900 participants completed a structured same-day patient satisfaction interview administered by trained research assistants who did not have access to clinician allocation, intervention status or the EMR interface. Interviews assessed participants’ perceptions of the consultation, including clinician communication, perceived thoroughness of assessment, clarity of explanations and overall satisfaction.

An independent expert panel of six family physicians (five female and one male), each registered with the Kenya Medical Practitioners and Dentists Council and with 10–16 years of clinical experience in Kenya, adjudicated all primary and clinical secondary outcomes. For adjudication of the primary outcome, panel members reviewed standardized summaries derived from follow-up data and clinical event information, with clinician allocation removed. Adjudicators did not have access to decision-support outputs for primary outcome classification. Two panel members independently reviewed each reported event to determine whether it met criteria for treatment failure and whether it was related to the original presentation. Discrepancies were resolved through discussion, and when consensus could not be reached, a third panel member served as arbiter.

### Sample size

The sample size calculation accounted for clustering at the clinical officer level and was powered to detect a 50% relative reduction in treatment failure within 14 days, from an expected failure proportion of 2% in the control arm to 1% in the intervention arm. Assuming a design effect of 1.5, 80% power, a two-sided alpha of 0.05 and 10% loss to follow-up, the target enrollment was 9,000 patient encounters, corresponding to 100 clinical officer clusters with a mean cluster size of approximately 90 encounters.

### Data collection, management and monitoring

Consultation data were recorded directly within the EMR at the point of care by the clinical officers. Follow-up data were collected using structured electronic case report forms administered by trained research assistants. Data quality was maintained through automated validation checks embedded within the EMR, routine data monitoring queries and periodic review by an independent monitoring team. All electronic data were stored on secure, password-protected servers with encrypted backups. Access was restricted through role-based permissions.

### Statistical analysis

The primary analyses followed the ITT principle at the level of the randomized clinician cluster, with participants analyzed according to the allocation of the treating clinician. Missing outcomes were handled using complete-case analysis given the low proportion of missing data. Protocol deviations were defined as encounters in which patients were managed by clinicians assigned to a different study arm than originally recorded, resulting in potential exposure misclassification. These encounters were excluded from per-protocol analyses but retained in the ITT analysis. A detailed classification of protocol deviations and violations is provided in the publicly accessible study repository^26^. For all analyses, random effects were used to enable clustering by clinical officer and facility. For binary outcomes (including the primary outcome), a mixed-effects logistic regression model was used to estimate the aOR, with its corresponding 95% CI. Ordinal secondary outcomes were analyzed using a mixed-effects proportion-odds logistic regression model. The risk difference was estimated by computing marginal predicted risks for each treatment group from the fitted logistic model and taking their difference, with corresponding credible intervals obtained using Bayesian multilevel logistic regression. A one-stage Bayesian multilevel individual participant data meta-analysis was used to estimate pooled and hospital-specific treatment effects while accommodating sparse data and clustering. Between-hospital heterogeneity was quantified through the random treatment-slope variance. Prespecified exploratory subgroup analyses examined potential effect modification by patient age group, presenting condition (sentinel versus nonsentinel) and consultation timing.

In post hoc analyses, we assessed the effect of the intervention on a composite outcome of hospitalization or death within 14 days, the per-patient cost of LLM use (calculated from tokens generated per consultation multiplied by the unit token cost and summarized as means with 95% CIs), consultation costs by medication category and consultation duration. Consultation costs were compared using multilevel linear regression with an interaction between study arm and drug category, and random effects for clinical officer and facility. Consultation duration was compared using the Wilcoxon rank-sum test, reporting medians, IQRs and the median difference. All post hoc analyses were labeled as nonconfirmatory and interpreted cautiously without adjustment for multiple comparisons.

Analyses were conducted in R (version 4.5.1). Reporting followed the Consolidated Standards of Reporting Trials (CONSORT)-AI extension for clinical trials evaluating AI interventions^42^ and the CONSORT 2010 statement: extension to cluster randomised trials^43^.

### Ethics and oversight

All data handling complied with the Kenya Data Protection Act (2019). Personal identifiers (including names, contact details and national identification numbers) were removed before model processing. The decision-support system operated within a secure clinical environment, and only de-identified clinical information required for generating recommendations was processed by the model. Role-based access controls were implemented within the EMR to restrict system access to authorized users. Data were retained in accordance with institutional data governance policies and study approvals. During the consent process, participants were informed that an electronic clinical decision-support tool incorporating AI would be used to assist clinicians during consultations. Participant information sheets and consent form templates are available alongside the study protocol^26^.

Adverse events and serious adverse events were monitored throughout the study using standardized procedures prespecified in the trial protocol. Suspected events were identified through follow-up assessments conducted by trained research assistants independent of the implementing healthcare organization, and internal review of clinical records where indicated. Treating clinicians retained full responsibility for patient management decisions, including escalation or referral where clinically indicated. An experienced study physician conducted the initial clinical review in consultation with the principal investigator as part of safety oversight and reporting procedures, after which cases were reviewed by the independent expert adjudication panel for final determination and attribution. All serious adverse events, including deaths, were reported to the study sponsor, ethics committees and the independent data and safety monitoring board (DSMB) within 48 h of investigator awareness, with a detailed follow-up report submitted within five working days (or seven calendar days) as additional information became available. The DSMB conducted scheduled safety reviews during the study, including an early review after enrollment of the first 1,000 participants, and had authority to recommend protocol modification, temporary suspension or termination if safety concerns arose. Because the intervention functioned as clinician-facing decision support rather than automated clinical management, formal efficacy stopping rules were not prespecified. Internal operational reviews conducted by the implementing organization did not influence outcome classification or causality determinations for the trial. Full details of safety monitoring and reporting procedures are provided in the published protocol, while a complete, de-identified listing of adverse events and serious adverse events observed during the trial is available in the publicly accessible study repository^26^.

### Ethics approvals

The study received ethics approval from the Amref Health Africa Ethical and Scientific Review Committee (P1817/2025), with additional authorization from Nairobi (NCCG/HWN/REC/752) and Kiambu (HRDU/PAA/04/2025) counties and from the National Commission for Science, Technology and Innovation (P/25/416731). The Kenyan medical device regulator (the Pharmacy and Poisons Board) determined that the product fell outside its oversight scope, and thus no local equivalent to an ‘investigational device exemption’ was submitted.

### Ethics and inclusion statement

This study was codesigned and implemented with local researchers and clinicians in Kenya, who were actively involved in study conception, protocol development, contextual adaptation of the intervention, participant recruitment, data collection, clinical evaluation, interpretation of findings and paper preparation. The research addressed a question of direct relevance to the local health system and was conducted within routine primary care settings serving the participating communities. The independent evaluation panel comprised six locally licensed Kenyan physicians with experience in the study context. Authorship reflects substantive contributions to the work, consistent with International Committee of Medical Journal Editors principles, with representation from investigators based in the study setting across study leadership, implementation, analysis and writing roles. Findings were shared in a dissemination workshop involving operational leadership within the implementing healthcare organization, Ministry of Health leadership and other local stakeholders to support service improvement and ensure continued local relevance and benefit.

### Reporting summary

Further information on research design is available in the Nature Portfolio Reporting Summary linked to this article.

## Online content

Any methods, additional references, Nature Portfolio reporting summaries, source data, extended data, supplementary information, acknowledgments, peer review information, details of author contributions and competing interests, and statements of data and code availability are available at 10.1038/s41591-026-04503-6.

## Supplementary information

Supplementary Information Supplementary Tables 1–4: clinical officer characteristics, patient satisfaction respondent characteristics, medication costs and adjusted medication cost analyses, as well as the CONSORT checklist.

Reporting Summary

## Data Availability

The study protocol and statistical analysis plan (describing all data elements generated, collected and curated) are available via Zenodo at https://zenodo.org/records/15788148 (ref. ^26^). Full EHR extracts are not publicly available owing to the need to protect participant privacy, in accordance with the ethical approval for this study. De-identified input (that is, clinician-recorded information in the EHR) and output (that is, LLM-generated guidance and traffic light color) pairs, as well as clinical outcomes, and the raw Likert scale scores for the 2,000 cases reviewed in depth by experts, together with the data dictionary and analytic code necessary to fully replicate the results of the study, will be deposited in a legally compliant, public repository (independent of the study team) within 12 months of publication to facilitate re-use. Before the public release, qualified researchers may request the abovementioned individual-level data for academic use from the study team. Requests should include a research proposal, a statistical analysis plan and a justification for data use, and can be submitted via email to A.A. (email: AAgweyu@kemri-wellcome.org) or B.A.M (email: bmateen@path.org). All requests will be reviewed by the Sponsor’s (PATH) Office of Research Affairs and the Amref Health Africa Ethical and Scientific Review Committee. Any fees for the review carried out by the latter, which will be duly communicated before initiation of the review, will be the responsibility of the party requesting access. Review of the proposals may take up to 2 months, and approved requests will be granted access via a secure platform after execution of a data access agreement.

## Code availability

AI Consult 2.0 (Penda Health) comprises three elements: (1) the cloud-based EMR system is a bespoke implementation (for Penda Health) of EasyClinic’s proprietary EMR solution, which includes integration of the LLM API, the construction of the ‘focus out’ event design that enables passive prompting of the LLM (codesigned with Penda Health) and the specific changes to the user interface to accommodate LLM outputs (as described in the paper, and codesigned with Penda Health), (2) the LLM instruction prompt and (3) the LLM. The LLM (GPT-4o, developed by OpenAI) is a proprietary product whose weights are not publicly available, and access was obtained via the commercially available API under an enterprise license. The product can be procured at will by any interested party, subject to the terms and conditions set out by OpenAI (the proprietor). The full instruction prompt is available without restriction available via Zenodo at https://zenodo.org/records/15788148 (ref. ^26^). Finally, full ‘demonstration’ build/test environments (comprising the EasyClinic EHR system integrated with the LLM and all rules) can be made available to third parties (not limited to research use) upon a formal request to R.K. (email: robert@pendahealth.com), subject to a suitable agreement (that is, testing by commercial entities or service providers looking to deploy will require different agreement structures to academics looking to verify study results). Requesting parties should provide a brief (1 page) description of their proposed use (and named users), which will be reviewed by Penda Health and EasyClinic to ensure feasibility of request, and confirm their willingness to cover the cost to the environment (US$700 as a one-off set-up fee, with a monthly hosting cost of up to US$500 (depending on the intensity of proposed use)). Penda Health and EasyClinic will provide this ‘demonstration build’ offering for up to 3 years from the date of publication. Full commercial use of the solution (comprising the EasyClinic EHR system integrated with the LLM and all rules) will need to be negotiated separately. Requests should be sent to R.K. (email: robert@pendahealth.com).
