# Supplementary material for: Generative AI-enabled clinical decision support system in primary care: a pragmatic, cluster-randomized trial
Source: Nat Med. 2026 Jun 26;32(8):3032–9. doi: 10.1038/s41591-026-04503-6 (PMC13472850; doi:10.1038/s41591-026-04503-6)
Supplement: Supplementary file 1 [file 41591_2026_4503_MOESM1_ESM.pdf]

nature medicine

Supplementary information https://doi.org/10.1038/s41591-026-04503-6

Generative AI-enabled clinical decision support system in primary care: a pragmatic, cluster-randomized trial

In the format provided by the authors and unedited

Contents

Supplementary Table 1: Clinical Officer Characteristics by Study Arm 2
Supplementary Table 2: Characteristics and Satisfaction Ratings of Survey Respondents 3
Supplementary Table 3: Mean Medication Costs (USD) Per-Patient 4
Supplementary Table 4: Adjusted Mean Differences in Per-Patient Medication Costs by Study Arm 5
CONSORT 2010 checklist of information to include when reporting a cluster randomised trial 6

Supplementary Table 1: Clinical Officer Characteristics by Study Arm

**Characteristic**	**Overall**<br>**N = 103**	**Control**<br>**N = 51**	**Intervention**<br>**N = 52**
Years of clinical experience			
2-5 years	27 (26%)	14 (27%)	13 (25%)
5-8 years	46 (45%)	21 (41%)	25 (48%)
8+ years	30 (29%)	16 (31%)	14 (27%)
Duration at Penda Heath			
<3 months	14 (14%)	5 (9.8%)	9 (17%)
3 -12 months	27 (26%)	15 (29%)	12 (23%)
12-23 months	13 (13%)	6 (12%)	7 (13%)
2-5 years	32 (31%)	16 (31%)	16 (31%)
5+ years	17 (17%)	9 (18%)	8 (15%)

Supplementary Table 2: Characteristics and Satisfaction Ratings of Survey Respondents

**Characteristic**	**Overall (N = 826)**	**Control (N = 411)**	**Intervention (N = 415)**
Age category: no. (%)			
< 1 year	30 (3.6)	11 (2.7)	19 (4.6)
1-5 years	161 (19)	78 (19)	83 (20)
5-18 years	115 (14)	55 (13)	60 (14)
18-54 years	514 (62)	263 (64)	251 (60)
≥ 55 years	6 (0.7)	4 (1.0)	2 (0.5)
Sex: no. (%)			
Female	476 (58)	243 (59)	233 (56)
Male	350 (42)	168 (41)	182 (44)
Perceived consultation length: no. (%)			
Too long	22 (2.7)	12 (2.9)	10 (2.4)
Just right	784 (95)	387 (94)	397 (96)
Too short	20 (2.4)	12 (2.9)	8 (1.9)
Patient-reported satisfaction: median (IQR)			
Next steps in management were clearly explained	4.0 (4.0-5.0)	4.0 (4.0-5.0)	4.0 (4.0-5.0)
Consultation was thorough and comprehensive	4.0 (4.0-5.0)	4.0 (4.0-5.0)	4.0 (4.0-5.0)
Concerns were adequately addressed	4.0 (4.0-5.0)	4.0 (4.0-5.0)	4.0 (4.0-5.0)
Would recommend this clinic to others	4.0 (4.0-5.0)	4.0 (4.0-5.0)	4.0 (4.0-5.0)

Supplementary Table 3: Mean Medication Costs (USD) Per-Patient

**Medication Category**	**Overall (N = 9,347), USD (SD)**	**Control (N = 4,654), USD (SD)**	**Intervention (N = 4,693), USD (SD)**
Antibiotics	3.78 (6.59)	3.85 (6.72)	3.71 (6.47)
Antimalarials	0.022 (0.446)	0.019 (0.426)	0.025 (0.464)
Antihistamines	0.895 (1.892)	0.920 (1.859)	0.872 (1.924)
Analgesics, antipyretics, anti-inflammatories	2.05 (2.34)	2.08 (2.35)	2.02 (2.32)
Respiratory medications (non-antibiotic, non-antihistamine)	0.224 (1.54)	0.192 (1.30)	0.256 (1.75)
Gastrointestinal medications	0.72 (2.35)	0.68 (2.26)	0.75 (2.43)
Chronic disease medications	0.14 (1.68)	0.15 (1.84)	0.13 (1.52)
Reproductive health, STI & genitourinary medications	0.099 (1.01)	0.094 (1.12)	0.104 (0.88)
Dermatological medications	0.36 (2.04)	0.33 (1.98)	0.39 (2.09)
Mental health & neurological medications	0.015 (0.34)	0.019 (0.33)	0.011 (0.36)
Vitamins & supplements	0.12 (0.75)	0.11 (0.73)	0.13 (0.77)
Miscellaneous & supportive medications	0.17 (0.84)	0.16 (0.79)	0.18 (0.90)

STI = Sexually transmitted infections
(1 USD = 130 Kenya Shillings)

Supplementary Table 4: Adjusted* Mean Differences in Per-Patient Medication Costs by Study Arm

Medication Category	Mean Difference in cost (USD)	95% CI
Antibiotics	−0.147	−0.252 to −0.042
Antimalarials	0.003	−0.102 to 0.108
Antihistamines	−0.051	−0.155 to 0.055
Analgesics, antipyretics, anti-inflammatories	−0.062	−0.167 to 0.043
Respiratory medications (non-antibiotic, non-antihistamine)	0.062	−0.043 to 0.168
Gastrointestinal medications	0.076	−0.029 to 0.182
Chronic disease medications	−0.022	−0.128 to 0.082
Reproductive health, STI & genitourinary medications	0.008	−0.097 to 0.114
Dermatological medications	0.053	−0.052 to 0.158
Mental health & neurological medications	−0.011	−0.116 to 0.095
Vitamins & supplements	0.021	−0.085 to 0.125
Miscellaneous & supportive medications	0.023	−0.082 to 0.128

*Adjusted for clustering within clinical officer and health facility

Mean difference calculated as: Intervention - Control (negative values indicate lower costs in the intervention arm)

STI = Sexually transmitted infections

(1 USD = 130 Kenya Shillings)

CONSORT 2010 checklist of information to include when reporting a cluster randomised trial

Section/Topic	*Item No*	*Standard Checklist item*	*Extension for cluster designs*	*Page No **
Title and abstract				
	1a	Identification as a randomised trial in the title	Identification as a cluster randomised trial in the title	**1**
	1b	Structured summary of trial design, methods, results, and conclusions (for specific guidance see CONSORT for abstracts)	See table 2	**2**
Introduction				
Background and objectives	2a	Scientific background and explanation of rationale	Rationale for using a cluster design	**3**
	2b	Specific objectives or hypotheses	Whether objectives pertain to the the cluster level, the individual participant level or both	**3**
Methods				
Trial design	3a	Description of trial design (such as parallel, factorial) including allocation ratio	Definition of cluster and description of how the design features apply to the clusters	**17**
	3b	Important changes to methods after trial commencement (such as eligibility criteria), with reasons		**N/A**
Participants	4a	Eligibility criteria for participants	Eligibility criteria for clusters	**17**
	4b	Settings and locations where the data were collected		**19**
Interventions	5	The interventions for each group with sufficient details to allow replication, including how and when they were actually administered	Whether interventions pertain to the cluster level, the individual participant level or both	**17**
Outcomes	6a	Completely defined pre-specified primary and secondary outcome measures, including how and when they were assessed	Whether outcome measures pertain to the cluster level, the individual participant level or both	**18**

	6b	Any changes to trial outcomes after the trial commenced, with reasons		**N/A**
Sample size	7a	How sample size was determined	Method of calculation, number of clusters(s) (and whether equal or unequal cluster sizes are assumed), cluster size, a coefficient of intracluster correlation (ICC or *k*), and an indication of its uncertainty	**18-19**
	7b	When applicable, explanation of any interim analyses and stopping guidelines		**N/A**
Randomisation:				
Sequence generation	8a	Method used to generate the random allocation sequence		**18**
	8b	Type of randomisation; details of any restriction (such as blocking and block size)	Details of stratification or matching if used	**18**
Allocation concealment mechanism	9	Mechanism used to implement the random allocation sequence (such as sequentially numbered containers), describing any steps taken to conceal the sequence until interventions were assigned	Specification that allocation was based on clusters rather than individuals and whether allocation concealment (if any) was at the cluster level, the individual participant level or both	**18**
Implementation	10	Who generated the random allocation sequence, who enrolled participants, and who assigned participants to interventions	Replace by 10a, 10b and 10c	
	10a		Who generated the random allocation sequence, who enrolled clusters, and who assigned clusters to interventions	**18**
	10b		Mechanism by which individual participants were included in clusters for the purposes of the trial (such as complete enumeration, random sampling)	**18**
	10c		From whom consent was sought	**17**

			(representatives of the cluster, or individual cluster members, or both), and whether consent was sought before or after randomisation	
Blinding	11a	If done, who was blinded after assignment to interventions (for example, participants, care providers, those assessing outcomes) and how		**18**
	11b	If relevant, description of the similarity of interventions		**17**
Statistical methods	12a	Statistical methods used to compare groups for primary and secondary outcomes	How clustering was taken into account	**19**
	12b	Methods for additional analyses, such as subgroup analyses and adjusted analyses		**19**
			Results	
Participant flow (a diagram is strongly recommended)	13a	For each group, the numbers of participants who were randomly assigned, received intended treatment, and were analysed for the primary outcome	For each group, the numbers of clusters that were randomly assigned, received intended treatment, and were analysed for the primary outcome	**4**
	13b	For each group, losses and exclusions after randomisation, together with reasons	For each group, losses and exclusions for both clusters and individual cluster members	**4**
Recruitment	14a	Dates defining the periods of recruitment and follow-up		**4**
	14b	Why the trial ended or was stopped		**4**
Baseline data	15	A table showing baseline demographic and clinical characteristics for each group	Baseline characteristics for the individual and cluster levels as applicable for each group	**15**
Numbers analysed	16	For each group, number of participants (denominator) included in each analysis	For each group, number of clusters included in each analysis	**4**

		and whether the analysis was by original assigned groups		
Outcomes and estimation	17a	For each primary and secondary outcome, results for each group, and the estimated effect size and its precision (such as 95% confidence interval)	Results at the individual or cluster level as applicable and a coefficient of intracluster correlation (ICC or k) for each primary outcome	**4-5**
	17b	For binary outcomes, presentation of both absolute and relative effect sizes is recommended		**4**
Ancillary analyses	18	Results of any other analyses performed, including subgroup analyses and adjusted analyses, distinguishing pre-specified from exploratory		**4-5**
Harms	19	All important harms or unintended effects in each group (for specific guidance see CONSORT for harms)		**5**
Discussion				
Limitations	20	Trial limitations, addressing sources of potential bias, imprecision, and, if relevant, multiplicity of analyses		**7-8**
Generalisability	21	Generalisability (external validity, applicability) of the trial findings	Generalisability to clusters and/or individual participants (as relevant)	**7-8**
Interpretation	22	Interpretation consistent with results, balancing benefits and harms, and considering other relevant evidence		**6-8**
Other information				
Registration	23	Registration number and name of trial registry		**2**
Protocol	24	Where the full trial protocol can be accessed, if available		**20**

Funding	25	Sources of funding and other support (such as supply of drugs), role of funders	**9**

* *Note: page numbers optional depending on journal requirements*
