# Supplementary material for: Generative AI-enabled clinical decision support system in primary care: a pragmatic, cluster-randomized trial
Source: Nat Med. 2026 Jun 26;32(8):3032–9. doi: 10.1038/s41591-026-04503-6 (PMC13472850; doi:10.1038/s41591-026-04503-6)
Supplement: Supplementary file 2 [file 41591_2026_4503_MOESM2_ESM.pdf]

nature portfolio

Corresponding author(s): Bilal A. Mateen

Last updated by author(s): May 20, 2026

Reporting Summary

Nature Portfolio wishes to improve the reproducibility of the work that we publish. This form provides structure for consistency and transparency in reporting. For further information on Nature Portfolio policies, see our Editorial Policies and the Editorial Policy Checklist.

Statistics

For all statistical analyses, confirm that the following items are present in the figure legend, table legend, main text, or Methods section.

n/a	Confirmed	
☐	☒	The exact sample size (n) for each experimental group/condition, given as a discrete number and unit of measurement
☐	☒	A statement on whether measurements were taken from distinct samples or whether the same sample was measured repeatedly
☐	☒	The statistical test(s) used AND whether they are one- or two-sided<br>*Only common tests should be described solely by name; describe more complex techniques in the Methods section.*
☐	☒	A description of all covariates tested
☐	☒	A description of any assumptions or corrections, such as tests of normality and adjustment for multiple comparisons
☐	☒	A full description of the statistical parameters including central tendency (e.g. means) or other basic estimates (e.g. regression coefficient) AND variation (e.g. standard deviation) or associated estimates of uncertainty (e.g. confidence intervals)
☐	☒	For null hypothesis testing, the test statistic (e.g. F, t, r) with confidence intervals, effect sizes, degrees of freedom and P value noted<br>*Give P values as exact values whenever suitable.*
☐	☒	For Bayesian analysis, information on the choice of priors and Markov chain Monte Carlo settings
☐	☒	For hierarchical and complex designs, identification of the appropriate level for tests and full reporting of outcomes
☐	☒	Estimates of effect sizes (e.g. Cohen's d, Pearson's r), indicating how they were calculated

Our web collection on statistics for biologists contains articles on many of the points above.

Software and code

Policy information about availability of computer code

Data collection

Consultation data were recorded directly in the electronic medical record system by clinicians across 16 Penda Health facilities. Clinical outcome follow-up data were collected by trained research assistants using electronic case-report forms implemented in REDCap. For study evaluation, an independent panel of trained local physicians reviewed and annotated clinical encounters using a standardized assessment rubric. No custom code was used for data collection. The AI Consult 2.0 (Penda Health), is comprised of three elements: (1) the cloud-based EMR system is a bespoke implementation (for Penda Health) of EasyClinic's proprietary EMR solution, which includes integration of the LLM API, the construction of the 'focus out' event design that allows for passive prompting of the LLM (co-designed with Penda Health), and the specific changes to the user interface to accommodate LLM outputs (as described in the manuscript, and co-designed with Penda Health); (2) the LLM instruction prompt; (3) the LLM. The LLM (GPT-4o, developed by OpenAI) is a proprietary product whose weights are not publicly available, and access was obtained via the commercially available API under an enterprise license. The product can be procured at will by any interested party, subject to the terms and conditions set out by OpenAI (the proprietor). The full instruction prompt is available without restriction in the public study repository: https://zenodo.org/records/15788148. Finally, full 'demonstration' build/test environments (comprising the EasyClinic EHR system integrated with the LLM and all rules) can be made available to third parties (not limited to research use) upon a formal request to R.K. (Robert Korom <robert@pendahealth.com>), subject to a suitable agreement (i.e., testing by commercial entities or service providers looking to deploy will require different agreement structures to academics looking to verify study results). Requesting parties should provide a brief (1 page) description of their proposed use (and named users), which will be reviewed by PendaHealth and EasyClinic to ensure feasibility of request, and confirm their willingness to cover the cost of the environment ($700 as a one-off set-up fee, with a monthly hosting cost of up to $500 [depending on the intensity of proposed use]). PendaHealth and EasyClinic will provide this 'demonstration build' offering for up to 3 years from the date of publication. Full commercial use of the solution (comprising the EasyClinic EHR system integrated with the LLM and all rules) will need to be negotiated separately. Requests should be sent to R.K. (Robert Korom <robert@pendahealth.com>).

Data analysis	All statistical analyses were conducted using R (version 4.5.1).

For manuscripts utilizing custom algorithms or software that are central to the research but not yet described in published literature, software must be made available to editors and reviewers. We strongly encourage code deposition in a community repository (e.g. GitHub). See the Nature Portfolio guidelines for submitting code & software for further information.

Data

Policy information about availability of data

All manuscripts must include a data availability statement. This statement should provide the following information, where applicable:

- Accession codes, unique identifiers, or web links for publicly available datasets
- A description of any restrictions on data availability
- For clinical datasets or third party data, please ensure that the statement adheres to our policy

The study protocol and statistical analysis plan (describing all data elements generated, collected and curated) are available in the public study repository: https://zenodo.org/records/15788148. Full electronic health record (EHR) extracts are not publicly available due to the need to protect participant privacy, in accordance with the ethical approval for this study. Deidentified input (i.e., clinician-recorded information in the EHR)-output (i.e., LLM-generated guidance and traffic light colour) pairs, as well as clinical outcomes, and the raw Likert scale scores for the 2000 cases reviewed in-depth by experts, together with the data dictionary and analytic code necessary to fully replicate the results of the study, will be deposited in a legally compliant, public repository (independent of the study team) within 12 months of publication to facilitate re-use. Prior to the public release, qualified researchers may request the aforementioned individual-level data for academic use from the study team. Requests should include a research proposal, a statistical analysis plan, and a justification for data use, and can be submitted via email to A.A. (Ambrose Agweyu <AAgweyu@kemri-wellcome.org>) or B.A.M (Bilal A. Mateen <bmateen@path.org>). All requests will be reviewed by the Sponsor's (PATH) Office of Research Affairs and the Amref Health Africa Ethical and Scientific Review Committee. Any fees for the review carried out by the latter, which will be duly communicated prior to initiation of the review, will be the responsibility of the party requesting access. Review of the proposals may take up to 2 months, and approved requests will be granted access via a secure platform after execution of a data access agreement.

Research involving human participants, their data, or biological material

Policy information about studies with human participants or human data. See also policy information about sex, gender (identity/presentation), and sexual orientation and race, ethnicity and racism.

Reporting on sex and gender	Sex (male/female), defined as biological sex, was routinely recorded in the electronic medical record and included in the study dataset. Gender identity was not collected. Sex was used to describe the study population, and participant characteristics are reported by sex in Table 1. Sex-stratified analyses were not performed, as the study intervention and primary outcomes were not hypothesised a priori to differ by sex.
Reporting on race, ethnicity, or other socially relevant groupings	No data on race, ethnicity, or other socially constructed groupings were collected or analysed.
Population characteristics	The study included patients of all ages presenting for routine outpatient care at 16 Penda Health primary care facilities in Nairobi and Kiambu Counties, Kenya. A total of 17,626 patients were screened for eligibility, of whom 9,702 were enrolled in the trial. The primary analysis included 4,654 control encounters and 4,693 intervention encounters. Baseline demographic and clinical characteristics were similar between study groups. Overall, 5,658 of 9,347 participants (61%) were aged 18–55 years, and 5,271 (56%) were female.
Recruitment	During the study period, all patients attending consultations led by participating clinical officers were screened for eligibility. Eligibility assessment, participant invitation, and written informed consent were conducted by trained research assistants prior to the consultation. All patients under 18 years of age attending for any reason were eligible, with adolescents aged 12–18 years providing assent in addition to guardian consent. Adults attending for non-acute or planned wellness visits (e.g. weight checks, vaccinations, or routine antenatal care), individuals unable to provide informed consent, those unlikely to be reachable for follow-up, and patients requiring emergency care at the time of screening were excluded.
Ethics oversight	The study was reviewed and approved by the Amref Ethics and Scientific Review Committee (ESRC) in Kenya (protocol P1817-2025).

Note that full information on the approval of the study protocol must also be provided in the manuscript.

Field-specific reporting

Please select the one below that is the best fit for your research. If you are not sure, read the appropriate sections before making your selection.

☒ Life sciences ☐ Behavioural & social sciences ☐ Ecological, evolutionary & environmental sciences

For a reference copy of the document with all sections, see nature.com/documents/nr-reporting-summary-flat.pdf

Life sciences study design

All studies must disclose on these points even when the disclosure is negative.

Sample size	The sample size calculation accounted for clustering at the CO level and was powered to detect a 50% relative reduction in treatment failure within 14 days, from an expected failure proportion of 2% in the control arm to 1% in the intervention arm. Assuming a design effect of 1.5, 80% power, a two-sided alpha of 0.05, and 10% loss to follow-up, the target enrolment was 9,000 patient encounters, corresponding to 100 CO clusters with a mean cluster size of approximately 90 encounters.
Data exclusions	All enrolled patient encounters were included in the primary intention-to-treat analysis. Encounters were excluded from the primary analysis if participants withdrew consent, were lost to follow-up before outcome assessment, or were found to have major protocol violations identified after enrolment, including ineligibility at screening or incomplete outcome data. The number and reasons for exclusions are reported in the CONSORT flow diagram.
Replication	This was a single, prospectively conducted pragmatic cluster-randomized controlled trial implemented across 16 primary care facilities. The intervention, eligibility criteria, outcomes, and analysis plan were pre-specified in the study protocol. No independent replication experiments were performed; however, the trial was conducted across multiple facilities and providers to enhance generalisability and robustness of findings.
Randomization	Randomization occurred at the provider (CO) level. Block randomization was implemented by the study statistician, with variable block sizes of 4, 6, or 8. COs remained assigned to their randomized study arm throughout the study period and across routine clinical shifts. Allocation was linked to individual user accounts within the EMR rather than to specific computers; thus, COs used the intervention or control workflow whenever they provided clinical care, irrespective of workstation. Patients were implicitly assigned to study arms based on the allocation of the CO managing their consultation, consistent with the cluster-randomized design.
Blinding	Blinding of clinical officers was not feasible because those allocated to the intervention arm interacted directly with the LLM-based clinical decision support system embedded within the EMR. Patients, research assistants responsible for follow-up and outcome assessment, and members of the independent expert adjudication panel were blinded to clinical officer allocation. Data analysts were blinded to study arm during primary analyses.

Reporting for specific materials, systems and methods

We require information from authors about some types of materials, experimental systems and methods used in many studies. Here, indicate whether each material, system or method listed is relevant to your study. If you are not sure if a list item applies to your research, read the appropriate section before selecting a response.

Materials & experimental systems

n/a	Involved in the study	
☒	☐	Antibodies
☒	☐	Eukaryotic cell lines
☒	☐	Palaeontology and archaeology
☒	☐	Animals and other organisms
☒	☐	Clinical data
☒	☐	Dual use research of concern
☒	☐	Plants

Methods

n/a	Involved in the study	
☒	☐	ChIP-seq
☒	☐	Flow cytometry
☒	☐	MRI-based neuroimaging

Plants

Seed stocks	Not applicable
Novel plant genotypes	Not applicable
Authentication	Not applicable
